# Performance of Cockcroft-Gault, MDRD, and CKD-EPI in estimating prevalence of renal function and predicting survival in the oldest old

**DOI:** 10.1186/1471-2318-13-113

**Published:** 2013-10-25

**Authors:** Jorien M Willems, Tom Vlasveld, Wendy PJ den Elzen, Rudi GJ Westendorp, Ton J Rabelink, Anton JM de Craen, Gerard J Blauw

**Affiliations:** 1 Department of Gerontology and Geriatrics, C-2-R, Leiden University Medical Center, PO Box 9600, 2300, RC,Leiden, The Netherlands; 2 Department of Hematology, Leiden University Medical Center, Leiden, The Netherlands; 3 Department of Public Health and Primary Care, Leiden University Medical Center, Leiden, The Netherlands; 4 Department of Nephrology, Leiden University Medical Center, Leiden, The Netherlands; 5 Department of Internal Medicine, Bronovo Hospital, The Hague, The Netherlands

**Keywords:** Mortality, Renal function, Oldest old, GFR equation, MDRD, CKD-EPI

## Abstract

**Background:**

The question for prevalence estimation and validation of the various eGFRs in old age is still under debate. To assess renal function with increasing age, we estimated mean eGFR, in subjects aged 20–85 years. Furthermore, we assessed prevalence of eGFR in a population-based sample of 85 year olds and investigated the performance of these eGFRs in predicting mortality in the oldest old.

**Methods:**

Renal function with increasing age was assessed in subjects aged 20–85 years from the Bronovo Study Cohort. We estimated prevalences of eGFRs and mortality risks in a population-based study of persons aged 85 years and older, the Leiden 85-plus Study. The GFRs were estimated by three different formulas.

**Results:**

After the age of 70 years, the C-G tended to give relatively lower eGFRs. An eGFR < 60 was found in 90% of the subjects aged 85 years as calculated by C-G, in 55% of the subjects using MDRD and in 68% of the 85 year old subjects as calculated by CKD-EPI. When renal function was <30 ml/min/1.73 m^2^, an increased mortality risk was observed by C-G (HR 1.9 (95% CI 1.1-3.3)), by MDRD (HR 3.5 (95% CI 1.8-6.7)), whereas by CKD-EPI significance was not reached (HR 2.4 (95% CI 0.9-6.4)).

**Conclusions:**

Our study demonstrates that in subjects above age 70, C-G gives lower estimates of renal function when compared to MDRD and CKD-EPI. Furthermore, prevalence of renal dysfunction (CKD stage 1–3) at age 85 years was highest for C-G (90%), lowest for MDRD (55%), and 68% for CKD-EPI. Moreover, we found that in subjects aged 85 years MDRD predicted mortality best.

## Background

Chronic kidney disease (CKD) coinciding with impaired renal function is predominantly a disease of the elderly and is associated with an increased risk for all cause and cardiovascular mortality, even after controlling for known risk factors [1,2]. Furthermore, renal impairment also affects safety of many common drugs used in older people. The glomular filtration rate (GFR) is considered to be the best overall reflection of renal function, but is not easily measured in daily practice. Therefore creatinine clearance is usually assessed by the classic Cockroft-Gault formula (C-G) and an estimate of glomerular filtration rate is often calculated by the Modification of Diet and Renal Disease equation (MDRD) [3,4]. More recently the Chronic Kidney Disease Epidemiology Collaboration equation (CKD-EPI) has been suggested as a more accurate estimate of eGFR [5], especially in the relative high ranges of eGFR (CKD stage 1 and 2) [6,7]. However, these measures have not been used to assess the impact of eGFR on truly estimating renal function in older populations, and they are not well validated in the elderly. Since it is difficult to measure GFR in a large population based group of old people, the question of prevalence estimation and validation of the various eGFRs in this age group is still unanswered.

Compared to the MDRD and CKD-EPI estimates of GFR, an important characteristic of the C-G formula is the inclusion of total body weight in the equation, as a reflection of muscle mass, the main determinant of creatinine generation [3]. With increasing age, body composition changes with decreasing muscle mass and increasing fat tissue as characteristic features, resulting in decreased lean body mass in very old age [8,9]. These age related changes might have important effects on creatinine clearance as calculated by the C-G formula in older individuals. In contrast to the classic C-G formula, the MDRD and CKD-EPI equations incorporates body surface area, resulting in eGFRs per 1,73 m^2^ body surface area.

Since it is virtually impossible to validate the various eGFRs in large population based samples of oldest old people by measuring GFR, for example by creatinine or inulin clearance, it is of great importance to determine the best measure of GFR in older people. First, to assess renal function with increasing age, we estimated mean creatinine clearance by C-G, and eGFR by MDRD and CKD-EPI equations, in 10 year age groups of subjects aged 20–85 years. Then, we estimated prevalence of eGFR in a population-based sample of 85 year olds and investigated the performance of these eGFRs in predicting mortality in the oldest old.

## Methods

### Study population

To investigate renal function as calculated by the C-G, MDRD and CKD-EPI equations, a cohort of subjects aged 20 to 85 years was used (study 1). This cohort was originally established to set reference values for laboratory measurements for various age categories in the Bronovo Hospital, The Hague, The Netherlands [10,11], a general hospital affiliated with Leiden University Medical Center, Leiden, The Netherlands. There were no inclusion criteria. Exclusion criteria were pregnancy, diabetes mellitus, use of oral contraceptives, vitamin- or iron supplements, and oral anti-coagulants. From all participants a venous blood sample was drawn. The Medical Ethical Committee of Bronovo Hospital accorded the study and all participants provided informed consent to study participation.

For studying prevalence of renal dysfunction based on the three formulas and their performance in predicting all cause mortality, the population of the Leiden 85-plus Study was used (study 2). The Leiden 85-plus Study is a population-based prospective follow-up study of persons aged 85 years and older. There were no selection criteria other than age. At baseline, all individuals were living in Leiden, The Netherlands. A total number of 599 subjects (response rate 87%) agreed to participate [12]. All participants were visited at their place of residence where interviews took place, height and weight measurements were done and venous blood samples were drawn. After inclusion all subjects were followed for mortality until February 2009. The Medical Ethical Committee of the Leiden University Medical Center (LUMC) approved the study and all participants provided informed consent for study participation.

### Laboratory measurements

All blood samples were collected in sterile EDTA tubes. Plasma creatinine concentrations were determined in the Bronovo Hospital (study 1) with Synchron LX-20, Beckman Coulter and in the LUMC (study 2) according to the Jaffe method using Hitachi 747, Tokyo, Japan.

### Creatinine clearance and glomular filtration rate equations

Three equations for renal function were used in our analysis (Table 1): the Cockcroft-Gault formula (C-G) [3], the four variable Modification of Diet in Renal Disease equation (MDRD) [4], and the Chronic Kidney Disease Epidemiology Collaboration equation (CKD-EPI) [5].

**Table 1:** Formulas of C-G, MDRD and CKD-EPI

	**Gender**	Serum creatinine*	**eGFR (ml/min/1.73 m**^**2**^**)**
Cockroft-Gault	Female	All	((140- age) × bodyweight/serum creatinine) × 0.85
	Male	All	(140- age) × bodyweight/serum creatinine
MDRD	Female	All	175 × (serum creatinine/88.4)^- 1.154^ × age^-0.203^ × 0.742
	Male	All	175 × (serum creatinine/88.4)^- 1.154^ × age^-0.203^
CKD-EPI	Female	≤ 62	144 × (serum creatinine/88.4/0.7)^-0.329^ × (0.993)^age^
	Female	> 62	144 × (serum creatinine/88.4/0.7)^-1.209^ x (0.993)^age^
	Male	≤ 80	141 × (serum creatinine/88.4/0.7)^-0.411^ × (0.993)^age^
	Male	> 80	141 × (serum creatinine/88.4/0.7)^- 1.209^ × (0.993)^age^

*Serum creatinine is stated in μmol/L.

*Abbreviations*: eGFR estimated glomerular filtration rate, MDRD modification of diet in renal disease formula, CKD-EPI chronic kidney disease epidemiology equation.

Because all our individuals were of the Caucasian race the multiplier factor for race was not applied.

### Chronic kidney disease

CKD was defined according to the K/DOQI staging [13]. CKD stage 3 has been sub-divided into 30–44 (stage 3a) and 45–59 ml/min/1.73 m^2^ (stage 3b) as there is evidence of graded increase in mortality risk (Table 2) [14,15].

**Table 2:** Stages of chronic kidney disease

**GFR**	**CKD stage**	**Description**
≥ 60	Stage 1 and 2	Kidney damage with normal or mildly decreased GFR
45–59	Stage 3a	Moderately decreased GFR
30–44	Stage 3b	Moderately decreased GFR
< 30	Stage 4 and 5	Severely decreased GFR or kidney failure

Adapted from K/DOQI clinical practice guidelines [13].

GFR is stated in ml/min/1.73 m^2^.

CKD stage 3 has been sub-divided into 30–44 (*stage 3b*) and 45–59 ml/min/1.73m^2^ (*stage 3a*) as there is evidence of graded increase in mortality risk [9,14].

### Mortality

Mortality data of the Leiden 85-plus Study (study 2), recorded between the start of the study, 1 September 1997, and 1 February 2009, were obtained from the municipal registry, which are publicly available. For the deceased participants the cause of death was obtained from Statistics Netherlands. We obtained permission to collect this data and only the primary cause of death on the death certificate was used in our analyses.

### Statistical analyses

Data are presented as number (percentages) for clinical characteristics and as median (interquartile range) for continuous parameters. The association between measures of renal function and mortality was analyzed with sex-adjusted Cox proportional hazard models. Differences in laboratory measurements between the different categories of eGFR as well as between sexes were determined by Mann–Whitney tests. SPSS software (version 16.0.1, SPSS Inc, Chicago, Ill) was used for statistical analyses. P-values lower than 0.05 were considered statistically significant.

## Results

### Creatinine clearance and eGFR in subjects aged 20–85 years (study 1)

**Figure 1:**
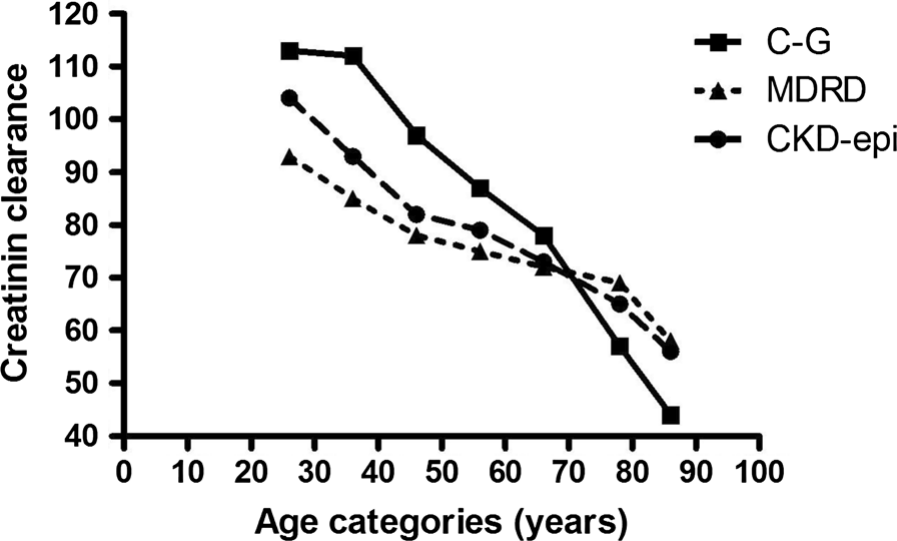
**Renal function in subjects of different age categories.** Modification of Diet in Renal Disease (MDRD) clearance and Chronic Kidney Disease Epidemiology Collaboration clearance (CKD-EPI) are expressed in ml/min/1.73 m^2^ and Cockroft-Gault (C-G) clearance in ml/min.

Characteristics of subjects in study 1 are reported in Table 3*.* The study sample comprised 242 subjects, 125 (52%) women and 117 (48%) men. In subjects aged 21 to 30 years, mean creatinine clearance as calculated by Cockroft-Gault formula was 117 ml/min, mean eGFR by MDRD formula was 94 ml/min/1.73 m^2^ and by CKD-EPI equation was 104 ml/min/1.73 m^2^. In the oldest age category, 71–85 years, creatinine clearance calculated by Cockroft-Gault formula was 57 ml/min, eGFR by MDRD formula 69 ml/min/1.73 m^2^ and by CKD-EPI equation 65 ml/min/1.73 m^2^. With increasing age, a significant decline of renal function was observed in C-G, MDRD and CKD-EPI (all p < 0.01). Figure 1 shows estimates of mean renal function as assessed with C-G, MDRD and CKD-EPI in the age categories. The three lines of the C-G, MDRD, and CKD-EPI cross at age 70 years. Before the age of 70 years, renal function as assessed by C-G is above the MDRD and CKD-EPI, indicating that before age 70 years eGFR assessed with the C-G formula tends to give relatively higher eGFRs. After the age of 70 years, the line of the renal function as assessed with the C-G formula is below the MDRD and CKD-EPI, indicating that after age 70 years, eGFRs assessed with the C-G formula tend to give relatively lower eGFRs.

**Table 3:** Characteristics of the Bronovo study cohort (study 1)

	**Age category (years)**					
	**21–30**	**31–40**	**41–50**	**51–60**	**61–70**	**71–85**
	**(n = 42)**	**(n = 43)**	**(n = 43)**	**(n = 52)**	**(n = 41)**	**(n = 21)**
Female, n (%)	23 (55)	22 (51)	23 (54)	25 (50)	23 (56)	9 (43)
Creatinine (μmol/L)	76 (71–87)	85 (74–91)	82 (72–97)	86 (74–99)	83 (74–93)	87 (76–97)
Cockroft-Gault (ml/min)	117 (97–127)	112 (95–138)	97 (82–112)	87 (77–96)	78 (68–85)	57 (52–82)
MDRD (ml/min/1.73 m^2^)	94 (85–102)	86 (77–92)	78 (72–87)	75 (66–84)	72 (66–82)	69 (58–77)
CKD-EPI (ml/min/1.73 m^2^)	104 (93–111)	93 (83–99)	82 (75–92)	78 (68–87)	71 (65–83)	65 (54–73)

Data presented are as median (IQR), unless otherwise stated.

*Abbreviations*: MDRD Modification of Diet in Renal Disease equation, CKD-EPI Chronic Kidney Disease Epidemiology Equation.

### Prevalence of renal dysfunction in the oldest old (study 2)

Characteristics of subjects in study 2 are reported in Table 4*.* Sixty-seven percent of the subjects was female and 30% had no comorbid illness. Median weight of all subjects was 69.5 kg and the median body mass index (BMI) was 26.7 kg/m^2^. Median creatinine clearance, calculated by C-G, was 43 ml/min. Using this formula, in 496/550 subjects (90%) a creatinine clearance lower than 60 ml/min was found. Furthermore, of these 85 years old subjects, the median eGFR, as calculated by MDRD, was 58 ml/min/1.73 m^2^ and 308/562 subjects (55%) were having an eGFR lower than 60 ml/min/1.73 m^2^. Moreover, when calculating GFR by CKD-EPI formula, a median eGFR of 53 ml/min/1.73 m^2^ was found and in 383/562 subjects (68%) eGFR was under 60 ml/min/1.73 m^2^.

**Table 4:** Characteristics of subjects aged 85 of the Leiden 85-Plus study

	**n = 562**
Female	377 (67)
Comorbid illness	398 (70)
Institutionalized	104 (18)
MMSE	26 (22–28)
Weight (kg)	69.5 (61.5–78.3)
Length (cm)	159 (154–166)
Body mass index (weigth/m^2^)	26.7 (24.2–29.9)
Body surface area (m^2^)*	1.72 (1.61–1.85)
Serum creatinine (μmol/L)	92 (81–108)
Cockroft-Gault (ml/min)∫	43 (37–51)
>60 ml/min	54 (10)
30–60 ml/min	454 (83)
<30 ml/min	42 (7)
MDRD (ml/min/1.73 m^2^)	58 (49–68)
>60 ml/min/1.73 m^2^	254 (45)
30–60 ml/min/1.73 m^2^	298 (53)
<30 ml/min/1.73 m^2^	10 (2)
CKD-EPI (ml/min/1.73 m^2^)	53 (46–62)
>60 ml/min/1.73 m^2^	179 (32)
30–60 ml/min/1.73 m^2^	365 (65)
<30 ml/min/1.73 m^2^	18 (3)

Continuous parameters are presented as median (IQR).

Categorial data are presented as number (%).

*Body Surface Area = calculated by DuBois’s formula.

∫ in 12 subjects weight was not available to calculate Cockroft-Gault clearance.

*Abbreviations*: MMSE Mini Mental State Examination, MDRD Modification of Diet in Renal Disease, CKD-EPI Chronic Kidney Disease Epidemiology Collaboration clearance.

**Figure 2:**
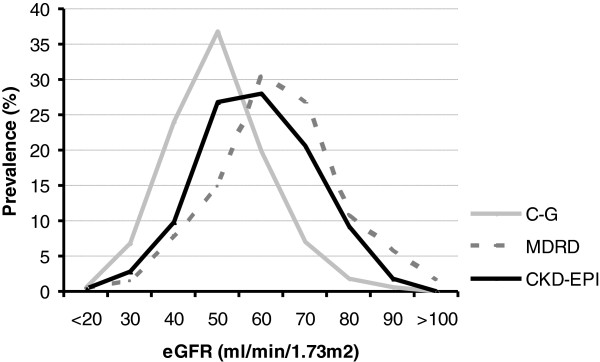
**Proportion of subjects in the various categories of eGFRs, calculated by C-G, MDRD and CKD-EPI.** Abbreviations: C-G; Cockroft-Gault formula, MDRD; Modification of Diet in Renal Disease, CKD-EPI; Chronic Kidney Disease Epidemiology Collaboration clearance.

We determined the proportion of subjects in the various categories of renal function, calculated by C-G, MDRD and CKD-EPI. Figure 2 illustrates that the C-G estimates result in higher prevalences of renal failure compared to MDRD and CKD-EPI.

Reclassification of subjects in the various eGFR categories assessed by MDRD and CKD-EPI is shown in Table 5. When the MDRD formula was used, 308/562 (54.8%) subjects were labelled as CKD stage 3 or worse (eGFR < 60 ml/min/1.73 m^2^). When the CKD-EPI formula was used, 383/562 (68.1%) subjects were classified as CKD stage 3 or worse. Overall, 92 subjects moved up one CKD stage when MDRD was used in stead of CKD-EPI. There were no subjects that moved up when CKD-EPI was used in stead of MDRD. The largest reclassification was for the subjects within the CKD-EPI category 30–59 ml/min/1.73 m^2^. Of the 365 subjects within this category, 75 were reclassified into the MDRD category 60–89 ml/min/1.73 m^2^.

**Table 5:** Number of subjects in categories of CKD assessed by MDRD and CKD-EPI

	**MDRD**					
CKD-EPI	<15	15-29	30-59	60-89	>90	
<15	**2**	0	0	0	0	2
15-29	0	**8**	*8*	0	0	16
30-59	0	0	**290**	*75*	0	365
60-89	0	0	0	**170**	*9*	179
>90	0	0	0	0	**0**	0
Total	2	8	298	245	9	562

Numbers in **bold** indicate those subjects who do not change CKD category on the basis of eGFR assessed with MDRD and CKD-EPI. *Italic* figures indicate the numbers of subjects who change into a different CKD stage.

MDRD and CKD-EPI clearance are stated in ml/min/1.73 m^2^.

*Abbreviations*: CKD Chronic Kidney Disease, MDRD Modification of Diet in Renal Disease, CKD-EPI Chronic Kidney Disease Epidemiology Collaboration clearance.

The associations between the different eGFRs and mortality risk are shown in Table 6. For the calculations of the mortality risk, eGFRs above 60 ml/min/1.73 m^2^ were set as reference group. With the C-G formula, an almost twofold increased risk for all-cause mortality was found for subjects with a renal function lower than 30 ml/min/1.73 m^2^ (HR 1.9 (95% CI 1.1-3.3)). An increased mortality risk was also observed by calculating renal function by MDRD, when renal function was under 30 ml/min/1.73 m^2^ (HR 3.5 (95% CI 1.8-6.7), and when renal function was between 30–44 ml/min/1.73 m^2^ (HR 1.6 (95% CI 1.2-2.2). When renal function was estimated with CKD-EPI, subjects with renal function lower than 30 ml/min/1.73 m^2^ had a similar increased all-cause mortality risk compared to MDRD, but significance was just not reached, HR 2.4 (95% CI 0.9-6.4).

**Table 6:** Relative mortality risks in categories of eGFR for C-G, MDRD and CKD-EPI

	**Hazard ratio (95% CI)**			
	< 30 ml/min	30-44 ml/min	45-59 ml/min	>60 ml/min
C-G	1.9 (1.1-3.3)*	1.3 (0.9-1.9)	1.0 (0.7-1.5)	1
MDRD	3.5 (1.8-6.7)**	1.6 (1.2-2.2)**	1.1 (0.9-1.3)	1
CKD-EPI	2.4 (0.9-6.4)	1.3 (0.9-1.8)	1.1 (0.9-1.5)	1

All hazard ratios are adjusted for sex.

C-G clearance is in ml/min, MDRD and CKD-EPI clearance is in ml/min/1.73 m^2^.

*Abbreviations*: C-G Cockcroft-Gault, MDRD Modification of Diet in Renal Disease, CKD-EPI Chronic Kidney Disease Epidemiology Collaboration clearance.

*P < 0.05, ** P ≤ 0.01 compared to the reference group (>60 ml/min).

## Discussion

The results of our study are threefold. First, our study demonstrates that in subjects under age 70 years, C-G gives higher estimates of renal function when compared to MDRD and CKD-EPI, while in subjects above age 70, C-G gives lower estimates of renal function when compared to MDRD and CKD-EPI. Second, prevalence of renal dysfunction (CKD stage 3–5) at age 85 years was highest for C-G (90%), lowest for MDRD (55%), and 68% for CKD-EPI. Third, we found that in subjects aged 85 years MDRD predicted mortality best. These results suggest that at very old age the MDRD formula might be the best estimate for eGFR, since the MDRD formula is most discriminative in predicting mortality.

### eGFR in various age categories

We showed that C-G, MDRD and CKD-EPI formula provide different estimates of renal function in various age categories. Under the age of 70, C-G clearance relatively overestimated renal function compared to eGFRs calculated by both MDRD clearance and CKD-EPI, whereas above the age of 70 creatinine clearance assessed with C-G formula resulted in relatively lower values. Our results are in line with earlier studies with older individuals (all mean age <85 years) [16,17], and also comparable with another community based study with younger participants (mean age of 75 years) [18]. The difference in mean age of the participants is the most plausible explanation between these study findings. The discrepancy in renal function calculated by the C-G formula and the MDRD and CKD-EPI equations may be explained by the intrinsic design of the estimates. In comparison with the MDRD and CKD-EPI equations, in the C-G formula the body weight is included next to the creatinine and age. In old age, lean body mass is reduced secondary to both sarcopenia and to increasing fat tissue [8,9]. The MDRD and CKD-EPI equations are adjusted for body surface area (BSA) [19], resulting in an eGFR value per 1.73 m^2^ BSA. Therefore, ageing may also have an effect on this adjusted eGFR since, next to the changes in body weight, both women and men loose height with increasing age, resulting in a decline in BSA in old age [20]. In the Leiden 85-plus Study mean BSA was 1.72 m2, suggesting that the BSA adjustment used in the MDRD and CKD-EPI estimates may, however, be appropriate in this very old population. It may be questioned whether it is appropriate to index the C-G formula for BSA, because weight is already included in the equation as a variable. Therefore, the use of the C-G formula in clinical practice for very old individuals is questionable.

### eGFR in the oldest old

Recently, the CKD-EPI, has been introduced in clinical practice, because of the possible inadequacies of the C-G and MDRD equations [4,5]. Our study shows that implementation of the CKD-EPI formula has consequences for very old subjects. Above the age of 70, eGFR calculated by CKD-EPI formula is underestimating renal function in comparison with eGFR calculated by MDRD, although not as much as the C-G formula. Compared with the classic C-G equation, introduction of the CKD-EPI formula will lower the amount of older individuals with CKD. Whereas based on these results, implementation of CKD-EPI formula would raise the number of older individuals with CKD on the basis of eGFR estimated with the MDRD formula, with as a consequence more hospitalizations, costs and also other therapeutic implications. A recently published large population based study of over a half million UK people of all ages [21] found that introduction of the CKD-EPI formula would reduce the prevalence of CKD in subjects < 70 years, but would raise the prevalence of CKD in the over 70 year old group. Furthermore, another report with particular emphasis of eGFR and the effect of age found that among the very elderly CKD-EPI may actually increase CKD prevalence estimates [22]. Although there are several studies with younger individuals that suggest that the CKD-EPI equation more accurately categorizes individuals [5,7] and although the US National Kidney Foundation has already recommended the adoption of the CKD-EPI formula for routine eGFR reporting by laboratories in the USA [23], based on our results and others [21,22,24-26], more research in the older individuals is warranted, before the CKD-EPI can be implemented in clinical practice for the oldest old age categories, in order to prevent unnecessary diagnostic procedures, therapeutic interventions and medical costs.

### Mortality risks and eGFR at old age

Since one goal of estimating renal function in clinical practice is to obtain estimates of deaths risk in various stages of CKD, it seems logical to use the equation that provides the best prediction of these outcome, especially in older individuals [1,2]. Therefore, we examined the association between the three different assessment methods for renal function and mortality in old age and found the MDRD equation to be best predictive for mortality. Subjects with MDRD < 45 ml/min/1.73 m^2^ had higher mortality risks compared to renal function calculatd by C-G or CKD-EPI formulae. Moreover, in subjects with creatinine clearance < 30 ml/min/1.73 m^2^ calculated by MDRD formula, a 3.5 increased risk of mortality was found. Our findings are in contrast with a large Italian study of 942 community dwelling subjects [18]. The participants of this study, the InCHIANTI study, had a mean age of 75 years. They found that only the C-G and not the MDRD equation was predictive for mortality. Since estimating equations C-G and MDRD both incorporate age in the formula, a plausible explanation for the discrepancy between findings of the two studies is the difference in mean age of the participants. In our study we only included oldest old subjects, all aged 85 years. However, a study with hospitalized older individuals in The Netherlands (mean age 78 year) showed results similar to ours [27]. Furthermore, in line with our findings, a large British cohort study of people aged 75 years and older, showed a increased mortality risk with MDRD < 45 ml/min/1.73 m^2^[15]. Data of very old community-dwelling very subjects (≥ 85 years) and the prediction of all cause mortality by C-G and especially MDRD and CKD-EPI formulae are scarce.

### Strong points and limitations

This is one of the few studies evaluating the effect of three different estimation methods for renal function in very old individuals in a population-based setting with a very high participation rate (87%) and with complete follow-up. This permits us to generalize our conclusions to very old people in the general population at large. Unfortunately, in both study cohorts, we did not have 24-hour urine collections for the measurement of creatinine clearance, although accuracy of urine collection at home done in a very old study population can be discussed. Moreover, accurate GFR measurements using inulin or iothalamate infusions are undoable for large scale study populations.

## Conclusions

In conclusion, estimation of renal function in very old persons can be facilitated by GFR equations, although C-G, MDRD and CKD-EPI all have their own limitations. We found that after age 70 years, C-G gives lower eGFRs and might therefore overestimating the number of individuals having CKD in comparison with both MDRD and CKD-EPI after the age of 70 years. Moreover, our results suggest that the MDRD formula might be the best estimate for eGFR in the oldest old followed by the CKD-EPI formula, since the MDRD formula is the best in predicting mortality. Our study suggests that implementation of CKD-EPI formula would raise the number of older individuals with CKD in comparison with the MDRD formula, with consequences for therapeutic decision making procedures and resulting in more referrals to nephrologists. Therefore, more research in older individuals is urgently needed, before the CKD-EPI can be implemented in clinical practice for the oldest old age categories.

## Competing interests

The authors declare that they have no competing interests.

## Authors’ contributions

JMW completed the statistical analysis, prepared the first draft of the paper, LTV revised the manuscript critically, WdE collected data, revised the manuscript critically, RGW provided oversight and consultation during all aspect of the study and revised the manuscript critically, TJR revised the manuscript critically, AJMdC performed research, checked the statistical analysis, and revised the manuscript critically; GJB designed research, provided oversight and consultation during all aspect of the study, and revised the manuscript critically. All authors read and approved the final manuscript.

## Pre-publication history

The pre-publication history for this paper can be accessed here:

http://www.biomedcentral.com/1471-2318/13/113/prepub

## References

[B1] Go AS Chertow GM Fan D McCulloch CE Hsu CY Chronic kidney disease and the risks of death, cardiovascular events, and hospitalization N Engl J Med 2004 351 13 1296 1305 10.1056/NEJMoa041031 15385656

[B2] Tonelli M Wiebe N Culleton B House A Rabbat C Fok M Chronic kidney disease and mortality risk: a systematic review J Am Soc Nephrol 2006 17 7 2034 2047 10.1681/ASN.2005101085 16738019

[B3] Cockcroft DW Gault MH Prediction of creatinine clearance from serum creatinine Nephron 1976 16 1 31 41 10.1159/000180580 1244564

[B4] Levey AS Coresh J Greene T Stevens LA Zhang YL Hendriksen S Using standardized serum creatinine values in the modification of diet in renal disease study equation for estimating glomerular filtration rate Ann Intern Med 2006 145 4 247 254 10.7326/0003-4819-145-4-200608150-00004 16908915

[B5] Matsushita K Tonelli M Lloyd A Levey AS Coresh J Hemmelgarn BR Clinical risk implications of the CKD epidemiology collaboration (CKD-EPI) equation compared with the modification of diet in renal disease (MDRD) study equation for estimated GFR Am J Kidney Dis 2012 60 2 241 249 10.1053/j.ajkd.2012.03.016 22560843

[B6] Levey AS Stevens LA Schmid CH Zhang YL Castro AF III Feldman HI A new equation to estimate glomerular filtration rate Ann Intern Med 2009 150 9 604 612 10.7326/0003-4819-150-9-200905050-00006 19414839 PMC2763564

[B7] Matsushita K Mahmoodi BK Woodward M Emberson JR Jafar TH Jee SH Comparison of risk prediction using the CKD-EPI equation and the MDRD study equation for estimated glomerular filtration rate JAMA 2012 307 18 1941 1951 10.1001/jama.2012.3954 22570462 PMC3837430

[B8] Beenakker KG Ling CH Meskers CG de Craen AJ Stijnen T Westendorp RG Maier AB Patterns of muscle strength loss with age in the general population and patients with a chronic inflammatory state Ageing Res Rev 2010 9 4 431 436 10.1016/j.arr.2010.05.005 20553969 PMC7105185

[B9] Doherty TJ Invited review: aging and sarcopenia J Appl Physiol 2003 95 4 1717 1727 12970377 10.1152/japplphysiol.00347.2003

[B10] Steen G Vlasveld LT Poot CC van der Slot-Verhoeven AJ Castel A Onderzoek naar referentiewaarden van laboratoriumonderzoek in een algemeen ziekenhuis: resultaten en bevindingen Ned Tijdschr Klin Chem Labgeneesk 2008 34 1 35 43

[B11] Willems JM Vlasveld LT Castel A Westendorp RG Blauw GJ Serum erythropoietin levels increase with age in healthy subjects Submitted 2013

[B12] der Wiel AB Van EE de Craen AJ Gussekloo J Lagaay AM Knook DL Westendorp RG A high response is not essential to prevent selection bias: results from the Leiden 85-plus study J Clin Epidemiol 2002 55 11 1119 1125 10.1016/S0895-4356(02)00505-X 12507676

[B13] National Kidney Foundation K/DOQI clinical practice guidelines for chronic kidney disease: evaluation, classification, and stratification Am J Kidney Dis 2002 39 2 Suppl 1 S1 S266 11904577

[B14] Coresh J Selvin E Stevens LA Manzi J Kusek JW Eggers P Prevalence of chronic kidney disease in the United States JAMA 2007 298 17 2038 2047 10.1001/jama.298.17.2038 17986697

[B15] Roderick PJ Atkins RJ Smeeth L Mylne A Nitsch DD Hubbard RB CKD and mortality risk in older people: a community-based population study in the United Kingdom Am J Kidney Dis 2009 53 6 950 960 10.1053/j.ajkd.2008.12.036 19394727

[B16] Pequignot R Belmin J Chauvelier S Gaubert JY Konrat C Duron E Hanon O Renal function in older hospital patients is more accurately estimated using the Cockcroft-Gault formula than the modification diet in renal disease formula J Am Geriatr Soc 2009 57 9 1638 1643 10.1111/j.1532-5415.2009.02385.x 19682124

[B17] Verhave JC Fesler P Ribstein J Du CG Mimran A Estimation of renal function in subjects with normal serum creatinine levels: influence of age and body mass index Am J Kidney Dis 2005 46 2 233 241 10.1053/j.ajkd.2005.05.011 16112041

[B18] Pizzarelli F Lauretani F Bandinelli S Windham GB Corsi AM Giannelli SV Predictivity of survival according to different equations for estimating renal function in community-dwelling elderly subjects Nephrol Dial Transplant 2009 24 4 1197 205 18988669 10.1093/ndt/gfn594 PMC2721425

[B19] DuBois D DuBois EF A formula to estimate the approximate surface area if height and weighth be known 1916 863 871 Ach Int Med. Ref Type: Generic 2520314

[B20] Holzenberger M Ruiz-Torres A Body surface area as a parameter of age decline Arch Gerontol Geriatr 1991 13 2 139 149 10.1016/0167-4943(91)90056-V 15374424

[B21] O’Callaghan CA Shine B Lasserson DS Chronic kidney disease: a large-scale population-based study of the effects of introducing the CKD-EPI formula for eGFR reporting BMJ Open 2011 1 2 e000308 10.1136/bmjopen-2011-000308 22184586 PMC3244664

[B22] Carter JL Stevens PE Irving JE Lamb EJ Estimating glomerular filtration rate: comparison of the CKD-EPI and MDRD equations in a large UK cohort with particular emphasis on the effect of age QJM 2011 104 10 839 847 10.1093/qjmed/hcr077 21652537

[B23] Becker BN Vassalotti JA A software upgrade: CKD testing in 2010 Am J Kidney Dis 2010 55 1 8 10 10.1053/j.ajkd.2009.11.005 20053344

[B24] Madero M Sarnak MJ Creatinine-based formulae for estimating glomerular filtration rate: is it time to change to chronic kidney disease epidemiology collaboration equation? Curr Opin Nephrol Hypertens 2011 20 6 622 630 10.1097/MNH.0b013e32834ba210 21941179

[B25] Chao CT Tsai HB Ko WJ Acute kidney injury in the elderly: only the tip of the iceberg J Clin Gerontol Geriatr 2013 in press (doi:10.1016/j.jcgg.2013.04.002)

[B26] Chao CT Wu VC Lai CF Shiao CC Huang TM Wu PC Advanced age affects the outcome-predictive power of RIFLE classification in geriatric patients with acute kidney injury Kidney Int 2012 82 8 920 927 10.1038/ki.2012.237 22763817

[B27] Rotmans JI Frenkel WJ Krediet RT de Rooij SE The predictive value of the Cockcroft-Gault formula and the modification of diet in renal disease formula for mortality in elderly people J Am Geriatr Soc 2009 57 5 946 948 10.1111/j.1532-5415.2009.02251.x 19470032

